# Imaging Features of Sclerosing Angiomatoid Nodular Transformation in Spleen

**DOI:** 10.1097/RCT.0000000000000910

**Published:** 2019-11-01

**Authors:** Jianbing Ma, Weiqiang Zhang, Lizhang Wang, Zefeng Zhu, Jia Wang, Jingfeng Zhang, Xiaofeng Yang

**Affiliations:** From the ∗ Department of Radiology, the First Affiliated Hospital, College of Medicine, Jiaxing University; † Department of Radiology, the First Affiliated of Hospital, College of Medicine, Zhejiang University, Zhejiang, P.R. China.

**Keywords:** sclerosing angiomatoid nodular transformation, spleen, CT, MR

## Abstract

The purpose of this study was to evaluate the features of sclerosing angiomatoid nodular transformation (SANT) in spleen on the imaging of computed tomography (CT) and magnetic resonance (MR).

**Materials and Methods:**

From July 2006 to April 2017, 12 patients with SANT confirmed by pathology were evaluated in a retrospective study. Eight patients were with CT imaging only, 2 patients were with MR imaging only, and 2 patients were with both CT and MR. Three professional senior radiologists analyzed the imaging features on CT and MR. The main characteristic analysis included size, margin, density, signal intensity, and enhancement pattern. The significant enhancement was defined as the degree of enhancement of lesion that is higher than the surrounding spleen parenchyma, and the mild enhancement was defined as the degree of enhancement of lesion that is lower than the surrounding spleen parenchyma.

**Results:**

All the 12 patients (5 men, 7 women; mean age, 45.8 years; age range, 21–62 years) presented as single lesion without special clinical symptoms. The range of lesions on diameter was from 25 to 80 mm. On CT images, 9 (90%) of 10 presented as hypodense in comparison with the parenchyma of spleen, 1 (10%) of 10 presented as isodense, and calcification was observed in 4 (40%) of 10 cases. On MR images, 4 (100%) of 4 manifested heterogeneous hypointensity on in-phase sequence and 3 (75%) of 4 performed as isointensity on out-of-phase sequence of T1-weighted. On the sequences of T2-weighted and diffusion-weighted image, 4 (100%) of 4 showed hypointensity. On CT and MR enhancement images, the number of significant enhancement and mild enhancement was 2 and 10, respectively. Seven (58%) of 12 showed progressive enhancement with the pattern of “spoke-wheel.”

**Conclusions:**

Imaging features on CT and MR have a high diagnostic value for SANT, especially when CT combined with MR examination.

Sclerosing angiomatoid nodular transformation (SANT) is a rare nonneoplastic vascular disease of spleen. It occurs more frequently in women with the average age of 50 years.^1,2^ The clinical symptoms are not specific, and most lesions are found occasionally by physical examination or in the treatment of other diseases.

Martel et al^1^ proposed the pathological name of SANT and described its detailed pathological and morphological features (solid mass, clear boundary, and multiple angiomatoid nodules) of SANT for the first time in 2004. Although angiomatoid nodule is the characteristic finding of SANT on pathology, it still has some overlap with other benign and malignant tumors of spleen such as hemangioma, lymphoma, splenic inflammatory pseudotumor, and littoral cell angioma. Therefore, it is very important how to distinguish SANT from these tumors on computed tomography (CT) and MR images.

Up to now, the features of SANT are mainly described on pathology,^3,4^ and there are few literatures that reported the imaging manifestations on CT or MR, including small series of case reports.^5–7^

Therefore, the aim of our study is to evaluate the imaging features of SANT on CT and MR with a largest series of cases up to now.

## MATERIALS AND METHODS

This study was approved by the Ethics Committee of the First Hospital of Jiaxing, and the requirement for individual consent was waived by the committee because of the retrospective nature of the study.

### Patients and Clinical Features

Twelve patients consisting of 7 women and 5 men (range, 21–62 years) were identified with pathologic diagnosis of SANT between July 2006 and April 2017 in our hospital and the First Affiliated Hospital of Zhejiang University, respectively. Left upper quadrant pain was found in 2 patients, 1 patient combined with splenic lymphoma, and the rest lesions were found by physical examination. Splenectomy was performed in all the patients.

### Image Acquisition

Because the machines were from different hospitals, scanning parameters were not totally consistent. Patients were asked to fast for 8 hours before the examination. Scan range was from diaphragmatic surface to bilateral renal hilum. All the 10 patients underwent CT plan and contrast-enhanced scan (ioversol), and the phases of enhancement included arterial (30–35 seconds), portal venous (55–60 seconds), and delayed phase (>120 seconds). Four patients underwent enhancement MR examination (gadolinium), the scan sequences included axial dual-echo T1-weighted, T2-weighted, and contrast-enhanced fat-saturated axial dynamic T1-weighted image (T1WI). Diffusion-weighted image (DWI) was obtained in 4 patients.

### Image Analysis

Computed tomography and MR images were separately analyzed on a workstation by 3 radiologists, and all the decisions were made in consensus. The following characteristics on CT and MR images were evaluated: (1) number, diameter, and location of lesions; (2) morphologic features of lesion including contour and border. (3) The calcification (high density, CT value was more than 100 Hounsfield unit) was recorded including the morphology and distribution. (4) The density was recorded as hypodense, isodense, or hyperdense compared with the surrounding normal splenic parenchyma. The signal intensity on T1WI (in phase and out-of-phase), T2-weighted image (T2WI), and DWI was recorded with the splenic peripheral parenchyma. (5) The signal intensity (as hypointense, isointense, or hyperintense) and density (as hypodense, isodense, or hyperdense) was recorded on CT and MR (arterial, portal venous, and delayed phase) images compared with the surrounding normal splenic parenchyma. (6) The homogeneity of each lesion was recorded as homogeneous or heterogeneous. (7) The enhancement pattern of each lesion on contrast-enhanced CT and MR images was recorded as progressive or wash-out and centripetal or centrifugal on delayed phase compared with arterial and portal venous phase was recorded. (8) Central scar was recorded on CT and MR imaging including the shape, density, signal, and the enhancement pattern.

### Pathologic Reviews

All the specimens were fixed with 4% neutral formalin and processed by routinely dehydrated, paraffin-embedded, and 5 μm sectioning. Hematoxylin eosin staining was used to confirm the diagnosis of SANT according to the criteria originated by Martel et al.^1^ Central scar, calcification, hemosiderin, angiomatous nodules, and inflammatory pseudotumor area were recorded. Immunohistochemistry was performed by Envision 2-step staining, and the antibodies included CD8, CD21, CD31, CD34, CD68, and SMA. The pathological results were given from 2 professional senior pathologists, and all the decisions were made in consensus.

## RESULTS

### Clinical and Pathologic Characteristics

The clinical characteristics of the 12 patients are presented in Table 1. Two patients presented with left upper quadrant pain. Nine cases were found by physical examination. One of the 12 lesions was found during the operation owing to splenic lymphoma. Laboratory tests were unremarkable.

**TABLE 1:**
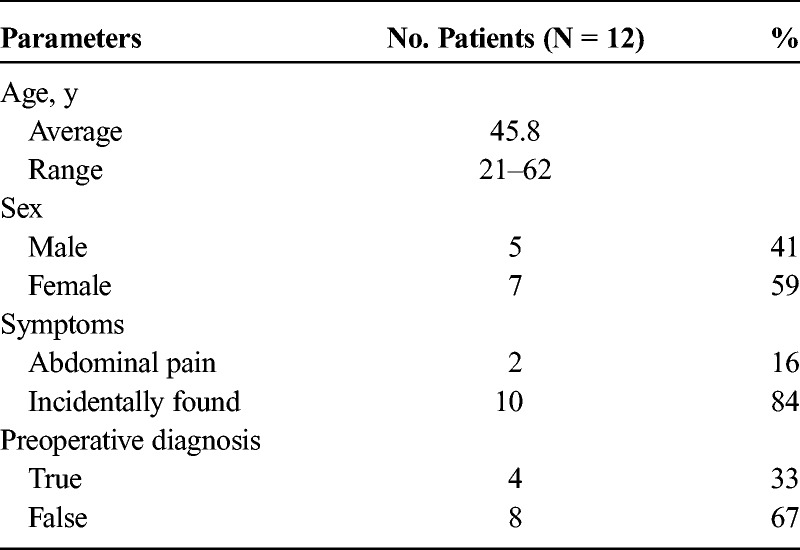
Clinical Characteristics of Patients With SANT

Pathologic characteristics of SANT of the spleen are presented in Tables 2 and 3.

**TABLE 2:**
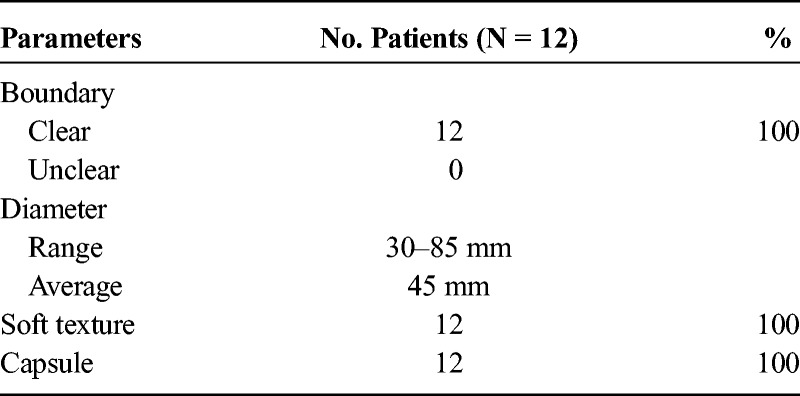
Gross Pathologic Characteristics of SANT of the Spleen

**TABLE 3:**
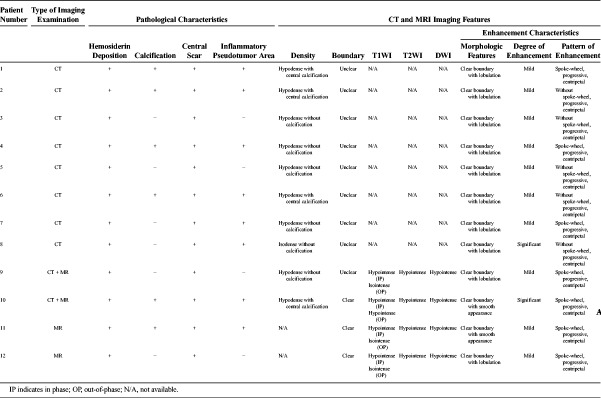
Comparative Analysis of Pathological Features and Imaging Manifestations

**FIGURE 1:**
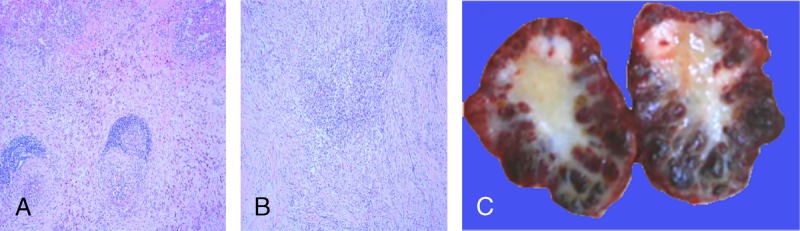
Sclerosing angiomatoid nodular transformation in a 49-year-old man. Photomicrograph (original magnification, ×100; hematoxylin [H] and eosin [E] stain) shows scattered circular or ovoid hemangiomatoid nodules with peripheral fibrous tissue arranged in concentric circles (A). Photomicrograph (original magnification, ×100; H and E stain) shows hemangiomatoid nodules surrounded by fibrous stroma (B); lymphocytes and plasma cells are seen in the stroma. On gross pathology image (C), the lesion is composed of numerous dark red nodules with the stellate yellow-white fibrous scar in the center.

Splenectomy was performed in each of the patient. All the 12 lesions presented as solitary masse with clear boundary, capsule, and soft texture. Typical gray-white stellate fibrous scars with peripheral reddish-brown nodules were found in all of them. Reddish-brown nodules were radially distributed around the lesion (Fig. 1C).

**FIGURE 2:**
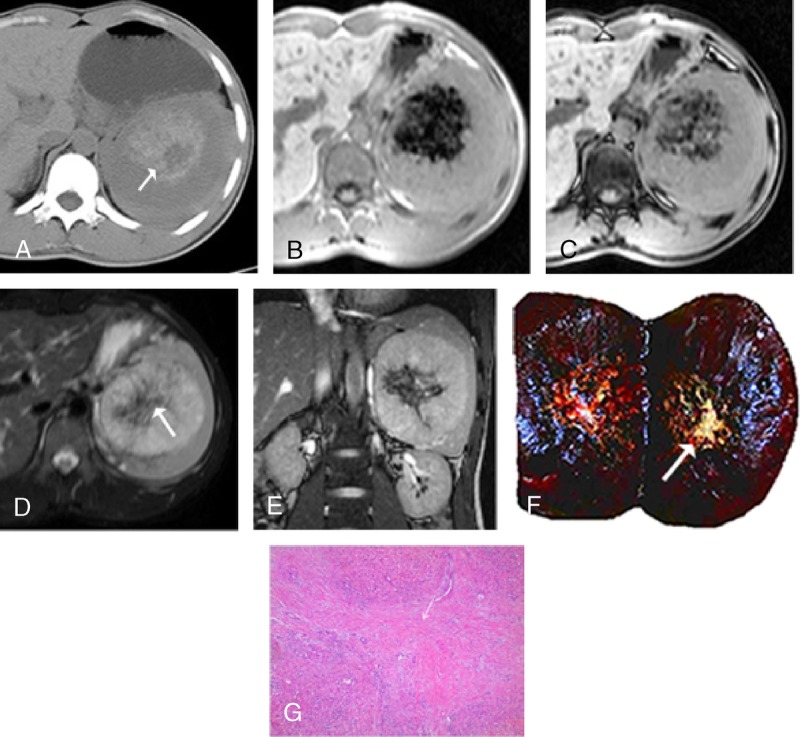
Sclerosing angiomatoid nodular transformation in a 21-year-old man. He was admitted to our hospital for left upper quadrant pain. Unenhanced axial CT image (A) shows a large solid lesion in spleen with the calcification of volcanic craters (arrow). T1-weighted (in- and out-of-phase) images (B, C) present heterogeneous hypointensity with clear boundary. Axial (D) and coronal (E) T2-weighted images show hypointensity with peripheral hyperintense lesion compared with the surrounding normal splenic parenchyma, with a stellate hypointense scar in the center. On gross pathology (F) image, an 11-cm well-defined lesion is seen with yellow stellate scar and calcification (arrow). Microscopic appearance (G), a large number of hyaline degeneration areas (arrow) are found.

Multiple angiomatous nodules and collagenized fibrous tissue were found in all the lesions (Fig. 1A). Angiomatous nodules were mainly composed of slit-like vessels lined with obese endothelial cells in the lumen. Fusiform and ovoid cells were seen around the slit-like vessels and arranged in target ring. Lymphocytes and plasma cells were seen in the stroma (Fig. 1B). The evidence of hemosiderin deposition was confirmed in all the lesions. Calcification was found in 6 lesions, and 1 of them presented as volcanic crater (Fig. 2F). In 8 lesions, inflammatory pseudotumor area was demonstrated (Fig. 2G). CD31, CD34, and SMA were positive in all lesions. CD21 was negative in all lesions. CD68 and CD8 were positive in 2 lesions.

#### CT and MR Findings

Results regarding the comparative analysis of imaging and pathology are summarized in Table 3. All the lesions showed single solid masses with range of 25 to 80 mm in diameter. The number of lesions located in the upper and lower poles of the spleen was 6, respectively.

**FIGURE 4:**
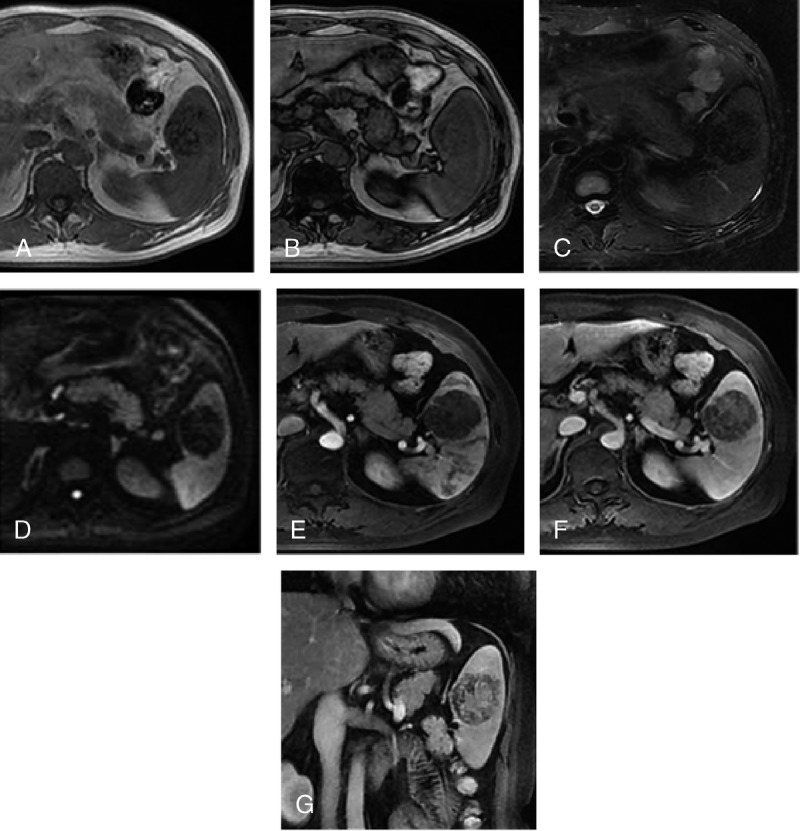
Sclerosing angiomatoid nodular transformation in a 49-year-old man. On the in-phase and out-of-phase images (A, B), the lesion presents heterogeneous hypointensity with ill-defined boundary. On axial T2-weighted and DWIs (C, D), the lesion shows remarkable hyperintense with clear boundary and lobulation. On the contrast-enhanced MR images including arterial (E), portal venous (F), and delayed phase (G), the lesion shows mild heterogeneous enhancement with the pattern of progressive and centripetal, and the typical sign of spoke-wheel is not found.

Density of lesions was hypodense in 9 (90%) of 10 cases (Fig. 2A) and isodense in 1 (10%) of 10 case. Nine (90%) of 10 lesions had unclear boundary. Calcification was present in 4 (40%) of 10 cases on CT images, and all of them were located in the center of the lesion, 3 of them showed as punctate; the other showed as volcanic craters (Fig. 2A). On T1-weighted (in phase) images, 4 (100%) of 4 appeared heterogeneously hypointense (Fig. 2B, Fig. 4A). On T1-weighted (out-of-phase) images, 1 (25%) of 4 appeared heterogeneously hypointense (Fig. 4B), and 3 (75%) of 4 appeared homogeneously isointense (Fig. 2C). On T2-weighted images, hypointense and hypointense with peripheral hyperintense appeared in 3 (75%) of 4 (Fig. 4C) and 1 (25%) of 4 cases (Figs. 2D, E), respectively. Whereas on DWI, 4 (100%) of 4 appeared hypointense (Fig. 4D).

**FIGURE 3:**
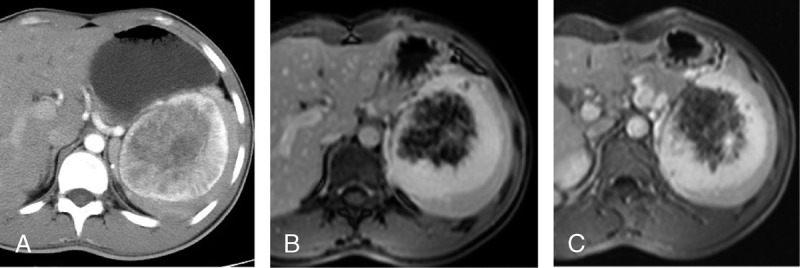
Sclerosing angiomatoid nodular transformation in a 21-year-old man. On the contrast-enhanced CT image (A), the lesion shows significantly enhancement with the pattern of spoke-wheel. On the images of contrast-enhanced MR (portal venous and delayed phase), the lesion shows a prominent enhancement with the pattern of progressive and centripetal, whereas no enhancement is seen in the center area (B, C).

Enhancement pattern of 10 (83%) of 12 lesions appeared as clear boundary with lobulation. On enhanced CT and MR images, the number of cases with mild and significant enhancement was 10 and 2, respectively. Seven (58%) of 12 lesions appeared typical feature of “spoke-wheel” (Figs. 3A, B, C), and all the lesions demonstrated progressive centripetal enhancement (Figs. 4E, F, G).

## DISCUSSION

Although the literatures concerning SANT of spleen appear more and more trend, most of them are about its pathological manifestations^3,4^ and there are little literatures reported on imaging features of SANT owing to its low incidence.^8,9^ Sclerosing angiomatoid nodular transformation (SANT) was formerly known as hemangioma,^10^ and it was also named multinodular hemangioma in the ninth edition of Rosai and Ackerman surgical pathology.^11^ Until 2004, it was officially named as sclerosing angiomatoid nodules transformation by Martel et al.^1^

Previous literatures reported that the rare disease mainly occurs in middle-aged people with a slight female preponderance and the mean age at onset was 50 years.^2,12^ The sex ratio in this study was 7:5, and the median age was 45.8 years, which were in line with the literatures.

The clinical symptoms of SANT are usually nonspecific, and most of them are found by physical examination or for the treatment of other unrelated diseases.^13,14^ In our present study, 9 (75%) of 12 patients were found incidentally during health check program or other disease. Two of them presented with left upper abdominal pain, and the other case was found by accident during in the treatment of lymphoma.

The etiology and pathogenesis of SANT are still unclear, and there are 2 main hypotheses at present. One hypothesis is that its occurrence is related to long-term stimulation of splenic red pulp. The other hypothesis is that SANT originated from a hemangioma,^1^ and it mainly goes through 2 stages in the early evolution process including nodular inflammatory pseudotumor formation and collagen fibrous tissue hyperplasia, and nodular hemangioma sample region was formed finally. Therefore, some of the lesions may have both inflammatory pseudotumor area and multiple angiomatoid nodules. In our study, the pathological mechanism is more similar to the latter and the similar phenomenon also occurred in eight cases. In addition, a large number of hyaline areas were also found in 1 of our cases, which were the basis for the formation of calcification.

In our study, there was no correlation between the diameter of the lesion and the patients age; in fact, the maximum and minimum diameters of the lesion were both depicted in young patients: in a 21- and 28-year-old man, respectively.

So far, no literature has reported recurrence or metastasis of the disease. Similarly, all the 12 patients were followed up for 3 years after treatment without associated chemotherapy or other therapy in our study, and recurrence or metastasis also was not found.

On unenhanced CT findings in our study, most of the lesions showed slightly hypodense compared with normal surrounding splenic parenchyma, and in 9 of 10 cases the lesions appeared unclear boundary. Calcification was found in 4 of the 10 cases, and 1 of them presented as “volcanic craters.” The lesions were slightly low density on CT imaging owing to the existence of the large number of fibrous tissues confirmed on pathology, and cystic degeneration or necrosis was not found. Similarly, in 3 of 10 cases, the lesions showed hypointensity in the T1WI; all the cases showed as hypointensity on T2WI and DWI, although the effect of hemosiderin deposition could not be completely excluded. In addition, although hemosiderin deposition was found in all our cases on histopathology, it was supported only in 2 of our cases in the sequence of chemical shift through observing the decrease in signal intensity on in-phase images compared with out-of-phase images. A similar pattern also was seen in the previous literatures.^9,15^ Hyaline degeneration was found in 8 of 12 cases, and calcification was present in 6 of 12 and 4 of 10 cases on pathology and CT images, respectively. Therefore, we speculated that there were some correlation between hyaline degeneration and calcification, which were as follows: (1) the form of calcification was related to the size of the hyaline area—the larger of the area of hyaline degeneration formed in the process of evolution, the more of the amount of calcification would be formed; (2) the morphology of calcification was related to the distribution of hyaline areas. The correlation between hyaline degeneration and calcification was also supported by previous pathological literature.^3^ In 1 of our cases with volcanic crater calcification, the large area of hyaline degeneration was also found on pathology. Hence, we believe that the different forms of calcification must be considered as one of the characteristic performance of the disease, even if it still is a rare finding.

On enhancement CT and MR imaging, most lesions manifested as mild or significant enhancement with clear boundary and lobulation. The pattern of enhancement was progressive and centripetal filling; the pathological basis of this feature was that hemangiomatous nodules were mostly located in the periphery of the lesion, and the large amount of fibrous tissue was located in the center of the lesion. In our study, all the lesions presented clear boundary, and in 10 of 12 cases, the lesions were lobulated. Seven of 12 lesions appeared typical sign of spoke-wheel, and it was consistent with the description of previous literatures.^16,17^ Among them, centripetal enhancement and wheel-spoke signs were considered as the characteristic manifestations of SANT, and these features helped to distinguish SANT from other vascular tumors of spleen.

### Differential Diagnosis

Most benign and malignant neoplasms of spleen should be distinguished from SANT, including hemangioma, lymphoma, splenic inflammatory pseudotumor, and littoral cell angioma.^18,19^ Hemangioma often appears as hypodense mass with clear boundary on CT images, and it shows an early marginal nodular enhancement in arterial phase and manifests centripetal progressive enhancement during portal venous and delayed phase. Although central scar is also found in some atypical lesions, signal intensity is not reduced on the T2-weighted sequence. Primary and secondary lymphoma of spleen is the most common malignant tumor; it usually presents as hypointense on T1-weighted sequence and hyperintense on T2-weighted sequence compared with the surrounding splenic parenchyma and shows mild enhancement. Splenic inflammatory pseudotumor is rare and benign; it presents as isointense or hypointense on T1-weighted sequence with well-defined margin, and it may show mild to moderate peripheral enhancement with the necrosis or fibrous tissue in the central area. Littoral cell angioma is not really rare, and the solitary lesion is easily confused with SANT. Although it also may show hypointensity on T2-weighted sequence owing to the hemosiderin deposition and centripetal progressive enhancement, the pattern of early enhancement is usually manifested as marginal nodular enhancement and the sign of spoke-wheel has not been reported up to now.

There were several limitations to our study. First, it is a retrospective analysis. Second, although it includes a small number of cases owing to the rarity of the mass, it represents the largest series imaged with CT and MR up to now. In addition, the machines and scanning parameters are not totally consistent owing to the different institutions.

## CONCLUSIONS

Sclerosing angiomatoid nodular transformation is a benign and rare vascular disease that usually affects the middle-aged women. At the same time, its clinical symptoms are not characteristic and surgical resection is the best treatment.

Our study, based on a largest series of SANT of the spleen so far, gives more imaging features of this vascular disease whose differential diagnosis still remains difficult owing to its rarity. However, the recognition of these imaging features on CT and MR is helpful for the accurate preoperative diagnosis of the disease.

As shown in our study, morphological features of calcification of the lesion can be clearly showed by CT examination, whereas MR examination has advantages in displaying certain image features, such as boundary, hemorrhage, necrosis, stellate fibrous scar, hemosiderin, and enhancement pattern. Therefore, CT combined with MR examination can improve the accuracy of preoperative imaging diagnosis of SANT. Among these imaging features, the sign of progressive enhancement with a spoke wheel pattern is considered to be the most typical manifestation for the diagnosis of SANT.
